# A Community Study of the Psychological Effects of the Omagh Car Bomb on Adults

**DOI:** 10.1371/journal.pone.0076618

**Published:** 2013-09-30

**Authors:** Michael Duffy, David Bolton, Kate Gillespie, Anke Ehlers, David M. Clark

**Affiliations:** 1 Queen’s University, Belfast, Northern Ireland; 2 Institute for Conflict Related Trauma, Enniskillen, Northern Ireland; 3 Cognitive Therapy Training, Dublin, Ireland; 4 University of Oxford, Oxford, United Kingdom; The University of Queensland, Australia

## Abstract

**Background:**

The main aims of the study were to assess psychological morbidity among adults nine months after a car bomb explosion in the town of Omagh, Northern Ireland and to identify predictors of chronic posttraumatic stress disorder symptoms.

**Method:**

A questionnaire was sent to all adults in households in The Omagh District Council area. The questionnaire comprised established predictors of PTSD (such as pre-trauma personal characteristics, type of exposure, initial emotional response and long-term adverse physical or financial problems), predictors derived from the Ehlers and Clark (2000) cognitive model, a measure of PTSD symptoms and the General Health Questionnaire.

**Results:**

Among respondents (n = 3131) the highest rates of PTSD symptoms and probable casesness (58.5%) were observed among people who were present in the street when the bomb exploded but elevated rates were also observed in people who subsequently attended the scene (21.8% probable caseness) and among people for whom someone close died (11.9%). People with a near miss (left the scene before the explosion) did not show elevated rates. Exposure to the bombing increased PTSD symptoms to a greater extent than general psychiatric symptoms. Previously established predictors accounted for 42% of the variance in PTSD symptoms among people directly exposed to the bombing. Predictors derived from the cognitive model accounted for 63%.

**Conclusions:**

High rates of chronic PTSD were observed in individuals exposed to the bombing. Psychological variables that are in principle amenable to treatment were the best predictors of PTSD symptoms. Teams planning treatment interventions for victims of future bombings and other traumas may wish to take these results into account.

## Introduction

Traumatic events trigger a wide range of emotional responses in individuals who are exposed to them. Posttraumatic stress disorder [1] a condition characterized by unwanted intrusive memories, avoidance of reminders and hyperarousal, is the most commonly discussed emotional sequel [2,3] However, other emotional responses including depression, anxiety and phobias also occur [4–6]. The prevalence of negative emotional responses varies with the type of trauma with rates being particularly high when intentional harm has been inflicted by others (bombings, rape, assaults, etc) [3,4,7–9]. For many people the negative emotional responses are relatively transitory with marked recovery being seen in the first few months after the trauma [3,10]. However, in a substantial sub-group the post-trauma symptoms persist and severely interfere with functioning [3,7]. In order to focus appropriate clinical resources on those who need help, it is important to understand which type of emotional reactions are most marked after potential traumas and what factors best predict in whom the reactions will become chronic. The present study aims to answer these questions using data from a community needs assessment that was conducted nine months after the Omagh Bomb. A particularly interesting possibility is that some of the factors that determine chronicity may be psychological variables that can be easily targeted in therapy and/or prevention programmes.

### The Omagh Bomb

On 15 August 1998, the deadliest single incident of Northern Ireland’s most recent period of conflict occurred when a car bomb exploded in Market Street in the centre of Omagh, a small market town with a population of 26,000 people. The Omagh bombing was unexpected in the context of the ongoing political process, coming just four months after the Belfast Agreement between the British and Irish Governments that provided a basis for a political settlement and reform. Misleading bomb warnings resulted in many people being moved to a perceived safe place just behind the car containing the explosive device. Thirty-one people, including two unborn children, were killed and 382 people were injured, of whom 135 were hospitalized. Twenty-six families were bereaved and of those killed, 15 were aged 17 years or under (*Source: Sperrin Lakeland Health & Social Care Trust* R*eport* 1998:*Strategy and implementation arrangements; meeting the needs arising from the Omagh bombing of the 15th August 1998*). The bomb had a devastating effect on the community. A large number of those injured were children and young people or mothers with young families. Many people sustained injuries resulting in the loss of limbs, loss of soft tissue, scarring and disfigurement. Many more who rushed to the scene to help witnessed scenes of intense horror and suffering.

In view of the large number of people exposed to potentially traumatic events and experiences associated with the explosion, the local Health and Social Care Trust decided to assess the impact of the bombing through a community needs assessment that would inform the service response. The needs assessment was conducted nine months after the bombing because research has shown that natural recovery is relatively modest if symptoms are still present at that time [3,10]. The assessment therefore focused on individuals for whom treatment was likely to be indicated.

In this study we report data from the needs assessment that addressed the following questions.

1 Does increasingly direct exposure to a potentially traumatic event increase PTSD symptoms to a greater extent than symptoms of general emotional distress (such as depression and anxiety)?
2 What individual and trauma characteristics predict chronic PTSD symptoms?
3 What are the emotional consequences of a “near miss” in terms of exposure to a potentially traumatic event?
4 Did individuals consider the extensive media coverage of the event a help or a hindrance for their attempts to come to terms with the event?

With respect to the first question, numerous studies have shown that PTSD rates are strongly influenced by the degree to which an individual is directly exposed to a traumatic event [6,11]. A number of studies have assessed both PTSD and other psychiatric problems. For example, Greiger et al. [12] assessed probable rates of PTSD and depression in Pentagon staff two years after the 11^th^ September 2001 terrorist attack and Freh and colleagues have assessed PTSD and co-morbidity following bombings in Iraq [13]. Being present at work at the time, seeing dead bodies and being injured all increased the rates of both PTSD and depression, compared to individuals who were not similarly exposed. The odds ratios for probable PTSD were higher than those for probable depression suggesting a stronger response in terms of PTSD symptoms. However, it could also be argued that the difference in diagnosis rates may be a function of differing severity thresholds. To avoid this problem, the present study reports standardized symptom severity scores, as well as probable caseness rates.

Turning to the second question, meta-analyses by Brewin et al [14] and by Ozler et al [15] have identified a range of predictors of PTSD. These include: pre-trauma personal characteristics (for example, previous emotional problems, less education, female), indices of trauma severity; and individuals’ responses at the time of the trauma (strong emotional reaction, dissociation). While each of these factors are significant predictors of PTSD among individuals exposed to potentially traumatic events, the amount of variability in PTSD that they explain either individually or together is modest. In an attempt to further improve prediction, Ehring, Ehlers and Glucksman [16,17] assessed a range of psychological factors specified in Ehlers and Clark’s [18] cognitive model of PTSD and compared them with the factors identified by Brewin et al [14] and Ozler et al [15] in a cross-sectional [16] and a prospective [17] study of PTSD following motor vehicle accidents. The cognitive factors were substantially more powerful in predicting PTSD. The present study investigates whether the same psychological factors may be similarly powerful in predicting chronic PTSD following a bombing. A positive result would have important therapeutic implications as the psychological factors can be targeted by psychological therapies.

Turning to the third question, for many potentially traumatic events there are people who could have been exposed but were not, either because they changed their plans at the last minute or left the scene shortly before the incident happened. Whilst there are studies examining the association of PTSD with different indices of exposure [11] as far as we are aware, there is no systematic research on the consequences of being a “near miss”. One could argue that people may be traumatized by knowing they could have been harmed. Alternatively, they could feel blessed or be not affected. A substantial number of people where in Market Street on the morning of the bombing but left shortly before the bomb went off and had no further direct exposure to the potentially traumatic events of that day. Comparing such individuals with those who had direct exposure and those who were not present at all that day allows us to determine the long-term emotional consequences of being a “near miss”.

A final question addressed by the community survey concerned peoples’ perceptions of the media response to the bombings and their overall perceptions of the effects of the bombings on community cohesion. The scale of the bombing and its unexpected nature given the ongoing political process meant that it attracted enormous media attention, including graphic TV broadcasts. There was concern that such coverage might be further traumatizing for members of the local community and other recent studies have identified media coverage to be associated with increased PTSD symptom levels [11,19,20]. There was a similar concern that the bombing may have adverse effects on the cohesion of the community.

## Methods

Ethical approval for the survey was granted by the Sperrin Lakeland Health & Social Care Trust which was the relevant ethical and institutional body at the time (1999). In addition to informing the local service response, the Trust anticipated that the findings of the Omagh study might be helpful to other communities experiencing such traumatic events. A specially developed questionnaire that focused on the Omagh bomb and its sequelae was sent to all adults living within The Omagh District Council area in May 1999. The purpose of the questionnaire was to explore adults’ reactions to the bombing and to help the public health and social care provider to respond to the needs of the local community. A week before the distribution of the questionnaire an explanatory flyer was circulated to every household to alert people to the study, encourage participation and explain that the findings of the study would be made available to other communities to help them deal with any future similar events. Residents who did not want their anonymized data to be used in this way could elect to ignore the survey. Approximately 34,000 questionnaires were distributed.

### The Questionnaire

Separate sections of the seven page questionnaire covered: pre-trauma personal characteristics and experiences (including prior emotional problems, previous mental health treatment, and prior history of traumatic events); degree of exposure to the bombing and related traumatic events (seeing the injured etc); emotional reaction at the time of the bombing or when the respondent heard about it; characteristics of their trauma memories; responses to the trauma memories; post-trauma beliefs; PTSD symptoms; general psychiatric symptoms; long-term physical or financial consequences of the bombing; the extent to which the bombing made respondents feel more or less part of the community; and the extent to which the media coverage was considered harmful or helpful (*Note: A copy of the questionnaire is available on request from the first author*).

Eleven items in the questionnaire covered exposure to various aspects of the trauma (see Table 1). Responses to these items were combined to create four mutually exclusive exposure categories. “In Market Street” means the respondent was in Market Street when the explosion happened. “Witness” means the respondent was not in Market Street at the time of the explosion but saw horrible scenes that day including people severely injured or dying. Most of these people would have come to help after the explosion. “Loss” means the respondent was not in Market Street at time of explosion or a witness but experienced loss of someone to whom they were close or loss of personal property. “Near miss” means the respondent was in Market Street shortly before the explosion but left, was not a witness and did not experience loss. “No exposure” means the respondent was not in Market Street that day, was not a witness, and did not experience loss.

**Table 1:** Types of Exposure Experienced by Respondents.

**Type of Exposure**	**No of Respondents**	**%**
*Questionnaire items concerning exposure to the trauma*		
In Market Street when explosion happened	118	3.8
Person injured	58	1.9
Person thought he/she was going to die	69	2.2
In Market Street shortly before bombing	522	16.7
In Market Street shortly after bombing	329	10.5
Person saw other people die	157	5.0
Person saw other people severely injured	455	14.5
Person saw horrible scenes	238	7.6
Someone close to person died	463	14.8
Someone close to person was injured	840	28.4
Lost property or job because of the bombing	34	1.1
*Exposure categories created from questionnaire responses*		
In Market Street when explosion happened	118	3.8
Witness	362	12.9
Loss	640	22.7
Near miss	218	7.7
No exposure	1476	52.5

PTSD symptoms were assessed by the Posttraumatic Diagnosis Scale (PDS) [21] a validated and widely used self-report measure of PTSD severity and probable PTSD caseness. The instructions explicitly mentioned the Omagh bomb. Respondents with a PDS score of 20 or more were considered probable PTSD cases, In two recent studies with 5 different samples this cut-off showed the best overall efficiency (0.88) in detecting PTSD following recent traumas [22,23]. Symptom cluster information alone performed less well than the overall score and did not further improve the prediction when added to the overall score. General psychiatric problems were assessed by the 12-item General Health Questionnaire (GHQ) [24] a well-validated and widely used self-report measure for assessing common psychiatric symptomatology and probable caseness in primary care. GHQ-12 items were scored in the conventional manner (0,0,1,1) with an overall score of 4 or more indicating probable casesness [24]. On both measures caseness indicates that these respondents meet probable diagnostic criteria for psychiatric disorder. The caseness cut-offs for both the PDS and the GHQ were chosen so they were among the most conservative that have been used in previous studies. Post-trauma beliefs were assessed by a shortened version of the Post-traumatic Cognitions Inventory (PTCI) [25] which has been shown to have good reliability and convergent validity and to discriminate between traumatized people with and without PTSD. A principal components factor analysis with varimax rotation identified two main PTCI factors in the survey population. Factor A, represented by 14 items, comprises negative beliefs about oneself and the symptoms of PTSD (e.g. “My reactions since the bombing mean I am going crazy”, “There is something wrong with me as a person”, “I can’t rely on myself”). Factor B, represented by 3 items, comprises beliefs about the world being an unsafe place (e.g. “You never know who will harm you”, “I have to be on guard all the time”).

Qualities of trauma memories were assessed by questions from previous research [26,27] and measured the disorganisation ("muddled, unclear") and perceived nowness ("seem to be happening now instead of being something from the past"). Responses to memories were assessed with shortened versions of the Response to Intrusions Questionnaire [28,29] assessing rumination (e.g., "I dwell on what life would have been like if the bombing had not happened") and suppression of thoughts and emotions (e.g., "I try hard to push them out of my mind").

### Respondents

Three thousand one hundred and thirty one questionnaires were returned, giving an overall response rate of approximately 10%. The mean age of respondents was 41.9 years (SD=16.5, range: 16-92), 62.8% were female, 60.7% were married or cohabiting, and 64% had children. The age profile is similar to that of the general adult population in the Omagh area but females are somewhat over-represented (females 50.31% in general population) (*Source: General Register Office (NI*)* 2001, 1999 mid-year population figures*)*.*

Table 2 shows how many respondents reported different types of exposure to traumas associated with the bombing. Four hundred and eighty six respondents had direct exposure to the horror of the bombing (either because they were in Market Street at the time of the explosion or were in the "Witness" group). This represents 37% of the police estimates of the total number of adults present in Market Street and surrounding areas when the bomb exploded. A further 640 respondents had indirect exposure in the sense that someone close to them died or was injured and/or they lost property as a consequence of the bombing- (the "Loss" Group).

**Table 2:** Caseness Rates According to the Posttraumatic Diagnosis Scale (PDS) and the General Health Questionnaire (GHQ).

**Type of Exposure**	**PDS Cases**					**GHQ Cases**				
	Yes	No	% Yes	OR	95% CI	Yes	No	% Yes	OR	95% CI
No exposure	42	1211	3.6			187	1210	13.4		
Near miss	8	181	4.2	1.26^*^	0.59-2.74	20	184	9.8	0.70^*^	0.43-1.14
Loss	68	503	11.9	3.90^*^	2.62-5.81	178	437	28.9	2.64^*^	2.09-3.33
Witness	72	259	21.8	8.02^*^	5.35-12.00	133	217	38.0	3.97^*^	3.04-5.17
In Market Street	62	44	58.5	40.63^*^	24.79–66.58	71	44	61.7	10.44^*^	6.96–15.68

Note: OR = odds ratio for probable caseness compared to the no exposure group. 95% CI = the 95% confidence interval for the OR estimate.

^*^ p<.001

### Statistical analysis

For most questionnaire items a proportion of respondents failed to complete the item. In the total sample missing data rates were below 7% for all relevant variables except for age (12%), years of education (12%), and PDS (14%). Among those who reported direct exposure (either in Market Street or as a witness of events) the missing data rates were generally lower, including those for age (8%) and PDS (9%). Each analysis used all available data.

In order to determine whether increasing exposure to the events connected with the bombing had a greater effect on PTSD symptoms than on general psychiatric symptoms, PDS and GHQ total scores were each converted to standard scores (mean = 0, SD = 1). The standard scores were then analysed with a mixed model two-way ANOVA with the between subjects factor being type of exposure and the within subjects factor being type of measure (PDS versus GHQ). For the probable casesness data Odds Ratios with respect to no exposure were compared for each exposure category.

Analyses of predictors of PTSD symptoms were restricted to those individuals who were directly exposed on the day (e.g. In Market Street or a Witness). First, correlations were computed between each potential predictor and PDS total scores at 9 months. Variables that were significant at this stage were carried forward to a second stage in which they were clustered into conceptually related groups and the overall amount of variance (adjusted R-squared) in PDS scores that each group explained was computed using multiple linear regression.

## Results

### Pre-Bomb Emotional Problems and Trauma Experience

Most respondents (83%) reported no or only slight emotional problems in the month prior to the bombing, 87.3% had not received previous help for anxiety or depression. Despite the prolonged conflict in Northern Ireland, almost 75% of the sample had not experienced a prior traumatic event.

### Symptoms and caseness as a function of type of exposure to the bombing

**Figure 1:**
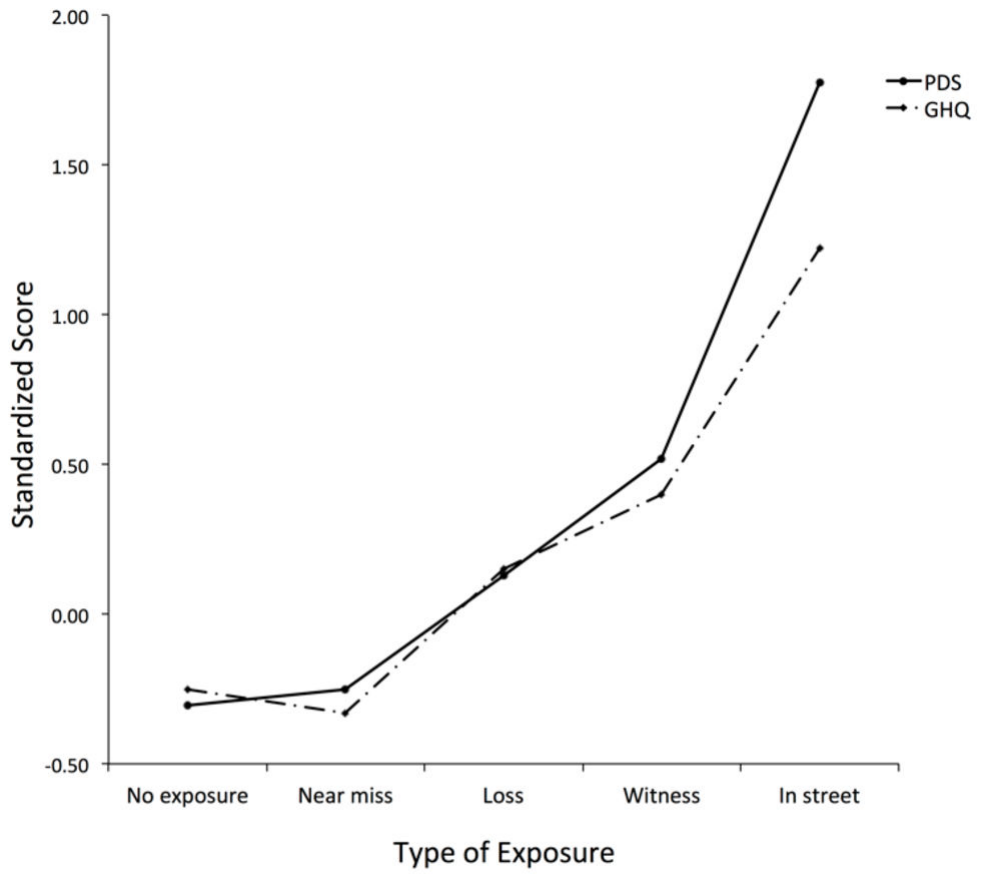
PDS and GHQ Standard Scores for types of exposure. A graphical representation of the Posttraumatic Diagnosis Scale (PDS) and General Health Questionnaire (GHQ) standardized symptom scores for each type of exposure to the Omagh Bombing.

Figure 1 shows the PDS and GHQ standardized symptom scores for each type of exposure. An ANOVA with the between-subjects factor type of exposure and the within-subjects factor type of symptoms revealed a significant main effect of type of exposure (F(4,2426 = 159.9, p<.001) and a significant interaction between exposure and symptom type ( F(4, 2426 = 17.99, p<.001). The interaction remained significant when age, gender and years of education were entered as covariates. Inspection of Figure 1 shows that direct exposure was associated with substantial increases in both types of symptoms but that the increase was larger for PTSD symptoms than for general psychiatric distress. Standardized PDS scores were significantly higher than standardized GHQ scores in individuals who were in Market Street (F(1,104) = 16.1, η_p_^2^ = .292, p <.001) or witnessed horrible events on the day of the bombing (F(1,327) = 2.3, η_p_^2^ = .017, p <.05). By contrast, for individuals who experienced Loss (F(1,566) = 0.3, η_p_^2^ = .001, p =.57) or a Near Miss (F(1,187) = 3.1, η_p_^2^ = .016, p =.08) there was no significant difference between the symptom types and for those with No Exposure, standardized PDS scores were significantly lower than standardized GHQ scores (F (1,1242) = 8.4, η_p_^2^ = .007, p< .01). For both the PDS and the GHQ, there was no significant difference between the No Exposure or the Near Miss group (PDS: F(1,1441)= 0.9, η_p_^2^ = .001, p=.35. GHQ: F(1,1441)= 2.2, η_p_^2^ = .001, p=.14) and symptoms scores for the Loss, Witness and In Market Street groups were significantly higher than those of either of these groups (all p's <.05). Among the direct and indirect exposure groups, the In Market Street group had significantly higher scores than the Witness group (p < .05) who in turn had significantly higher scores than the Loss group (p<.05).

Probable caseness rates (see Table 2) showed a similar pattern of results to standardized symptom scores. In particular, odds ratios compared to No Exposure increased with greater direct exposure, as one moves from the Loss group to the Witness group and then the group of people who were in Market Street when the bomb exploded. Although this effect was evident for both PDS and GHQ caseness, it was much more marked for the former. Among people who were in Market Street the OR for probable PDS casesness is 40.63, compared to 10.44 for probable GHQ casesness. Similarly among Witnesses, the OR for probable PDS caseness is 8.02, compared to 3.97 for probable GHQ caseness. The absolute rates of probable PDS caseness are 58.5% for people who were in Market Street and 21.8% for Witnesses.

### Predictors of PTSD symptoms among those who were directly exposed

Table 3 shows the correlations between each potential predictor and PDS scores among those individuals who were directly exposed (In Market Street or Witness). Table 4 shows the significant predictors that were entered into multiple regressions to estimate the proportion of variance (adjusted R-squared) in PDS scores explained by each of the conceptual categories highlighted in previous meta-analyses and the psychological factors highlighted in Ehlers and Clark’s [18] cognitive model of the maintenance of PTSD. Each of the categories highlighted in previous meta-analyses were significant predictors (p < .001) in multiple regression analyses but the amount of variance explained by each is modest: pre-trauma variables (5%), type of exposure (27%), individual’s emotional reaction at the time (24%) and bombing-related long-term adverse physical or financial problems (18%). The cognitive model variables account for substantially more variance (63%). To further examine the relative importance of the cognitive and other variables, we extracted each of the variables that were significant within the non-cognitive model categories and entered them into a single multiple regression. The eleven variables were: gender, years of education, emotional problems in the month before the bombing, being in Market Street at the time, being injured, seeing people die, emotional response at the time, thinking you would die, feeling part of the community, long-term physical health problems and long-term financial problems. Together these account for 42% of variance in PDS scores, still considerably less than the 63% accounted for by the six cognitive model variables, which are: rumination, thought-emotion suppression, nowness of the memory, a muddled memory, negative beliefs about oneself and the symptoms of PTSD (PTCI- Factor A), and beliefs about the world being an unsafe place (PTCI- Factor B). Combining the variables from previous meta-analyses and the cognitive model variables accounted for 72% of the variability in PTSD symptom scores.

**Table 3:** Correlations with Symptom Scores on the PDS and GHQ in Individuals with Direct Exposure (In Market Street at Time of Bombing and/or Witnessed Related Traumatic Events).

**Predictor**	**PDS**	**GHQ**
Seeing other people severely injured	-.01	-.02
Religious conviction	.02	-.02
Married or cohabiting	.03	.06
Having children	.04	.11^+^
Age	.07	.09
Lost property because of bombing	.09	.10^+^
Female Gender	.11^+^	.07
Emotional problems in 4 weeks before the bombing	.14*	.13*
Someone you are close to died	.15*	.12^+^
Perceived helpfulness of media coverage	-.15*	-.16**
Education (years)	-.16**	-.10^+^
Previous traumatic events	.17**	-.08
Previously received treatment for anxiety or depression	.17**	.20*
Feel more a part of Omagh Community since the bombing	-.20**	-.18**
Someone you are close to injured	.22**	.21**
Changed appearance as consequence of bombing	.22**	.14*
Physical handicap as a consequence of bombing	.23**	.12^+^
Seeing horrible scenes	.26**	.26**
Long-term financial problems from bombing	.27**	.17**
Seeing other people die	.31**	.23**
*Emotional response at the time*	.34**	.22**
Injured (self)	.39**	.29**
*PTCI Factor B (Unsafe World)*	.39**	.33**
In Market Street at the time of the explosion	.40**	.27**
Long term physical health problems arising from the bomb	.41**	.37**
Thought you were going to die at the time	.42**	.28**
Memories are muddled/unclear	.43**	.36**
*Thought-Emotion Suppression*	.45**	.27**
*Rumination*	.46**	.37**
Memories have “here & now” quality	.56**	.46**
*PTCI Factor A (Negative View of Symptoms and Self)*	.72**	.72**

Note. + denotes p<.05. * denotes p <.01 ** denotes p<.001. Factors in italics are multi-item scales. PTCI = Posttraumatic Cognitions Inventory

**Table 4:** Predictors of PTSD Symptoms at 9 months after the Explosion among People who were in Market Street at the Time and/or Witnessed Related Traumatic Events.

Variable	β	p	adjusted R^2^
*Pre-trauma personal variables*			.05
Age	.066	.195	
Female gender	.103	.038	
Education (years)	-.149	.003	
Emotional problems (in 4 weeks before bombing)	.144	.004	
Previous trauma	-.092	.070	
*Type of exposure*			.27
In Market Street	.202	<.001	
Injured	.224	<.001	
Saw people die	.177	<.001	
Saw people injured	.039	.369	
Saw horrible scenes	.083	.072	
Someone you are close to died	.150	<.001	
*Reactions at the time*			.24
Emotional response	.265	<.001	
Thought would die	.368	<.001	
*Long-term adverse physical or financial problems*			.18
Physical health	.342	<.001	
Physical handicap	.019	.703	
Changed appearance	.047	.343	
Financial problems	.116	.019	
*Cognitive model predictors*			.63
Rumination	.092	.018	
Thought-emotion suppression	.178	<.001	
Nowness of memory	.159	<.001	
Muddled memory	.098	.006	
PTCI - Factor A	.523	<.001	
PTCI - Factor B	-.009	.815	
*Social support*			.04
Married/cohabiting	-.016	.743	
Feeling part of community	-.200	<.001	

Note: PTCI = Posttraumatic Cognitions Inventory. PTCI- Factor A comprises negative beliefs about oneself and the symptoms of PTSD. PTCI- Factor B comprises beliefs about the world being an unsafe place.

### Perception of Media Coverage

Respondents’ views of the media coverage showed considerable variability but were mostly on the positive side. Considering all respondents, 67% regarded the media as helpful in relation to coming to terms with the event (‘slightly’ 24.2%, ‘moderately’ 24.4%, ‘very’17.9%) whilst 17.7% responded that the media was "no help" and 5.6% thought that the media was "harmful". Seventy-seven per cent regarded the media as helpful in relation to "explaining the impact" of the bombing (‘slightly’ 18.5%, ‘moderately’ 29.5%, ‘very’ 33.9%) compared to 11% who considered the media were ‘unhelpful’ with 7% responding that the media were harmful in this respect. Ninety percent found the media helpful in relation to "providing information about where to get help" (‘slightly’ 19.9%, ‘moderately’ 28.1%, ‘very’ 42%) whilst 2.9% responded that the media were ‘not helpful’ in this regard. To determine whether people who were directly exposed held different views about the media from people who were not directly exposed, responses were grouped into three categories (harmful, no help, helpful) and compared. People who were directly exposed were less positive in their views about the value of the media for coming to terms with what happened (χ^2^ = 28.7, df = 2, p <.001) and for explaining the impact of the bombing (χ^2^ = 40.8, df = 2, p <.001). Among the directly exposed, 57% thought the media coverage was helpful for coming to terms with what happened and 9.3% thought it was harmful. For explaining the impact of the bombing, 71.7% thought the media was helpful and 11.7% thought it was harmful.

### Effect of the Bombing on Views of the Community

Considering all respondents, many (40.6%) reported that they felt “more a part of the community" since the bombing, over half (53.2%) reported that they felt “no different” and only a small proportion (6.2%) indicated that they felt “less a part of the community" since the bombing. The views of people who were directly exposed on the day differed significantly from those who were not directly exposed (χ^2^ = 28.7, df = 2, p <.001), although the differences were small. Among the directly exposed, 45.1% felt “more a part of the community”, 44.5% felt “no different” and 10.4% felt “less a part of the community”. Direct exposure therefore appeared to polarize people’s views with more people endorsing feeling “more a part of the community” and more people endorsing feeling “less a part of the community”.

## Discussion

The first aim of the study was to determine the extent of negative emotional reactions in the community as a function of individuals’ exposure to the events of surrounding the Omagh bomb. For both PTSD (measured by the PDS) and general psychiatric problems (measured by the GHQ) average symptom levels and numbers of individuals who met caseness criteria were significantly higher in individuals who were exposed to the trauma in any manner than in individuals with no exposure. The highest symptom and caseness levels were shown by those who were in Market Street when the bomb went off (58.5% probable PTSD caseness), followed by those who saw horrible events on the day but were not present during the explosion (Witness group: 21.8% probable PTSD caseness), with those who had someone to whom they were close die or who lost property having lower, but still elevated levels (Loss group: 11.9% probable PTSD caseness). Little is known about the psychological consequences of being a near miss. The present data suggests that the impact is minimal. People who were in Market Street but left shortly before the bomb went off and were not otherwise exposed to the horrors of that day did not differ in symptom levels or caseness rates from individuals who had no exposure at all. It is generally accepted that exposure to trauma markedly increases levels of general psychiatric distress as well as the specific symptoms PTSD [6,30–32]. Our findings confirmed this broad symptom effect but also demonstrated that direct exposure to trauma has a somewhat greater effect on PTSD symptoms than on other psychiatric symptoms. We converted PDS and GHQ scores standard scores so they could be meaningfully compared. PDS standard scores were more elevated than GHQ standard scores among people who were present at the time of the explosion (In Market Street group) and among those who subsequently witnessed horrible events. Caseness odds ratios (ORs) compared to individuals with No Exposure showed a similar pattern. Among people in Market Street the OR for probable PTSD caseness was 40, compared to 10 for probable GHQ caseness.

In contrast to the findings for the In Market Street and Witness groups, the Loss group (who had experienced the loss of a significant other and/or property) showed similar increases in PTSD and other psychiatric symptoms. This further confirms the specificity of PTSD symptoms to trauma exposure, rather than negative events per se. Our overall findings are broadly consistent with findings from other large community trauma studies such as the New York Sept 11 terrorist attacks [6,11]. In those studies the prevalence of PTSD was higher amongst individuals with direct trauma exposure [6,11]. Galea et al [6] also found that material losses (job/possessions) substantially increased the rates of both PTSD symptoms and depression. However, they found that loss involving death of a significant other was more specifically linked to depression.

The second aim of the study was to identify predictors of chronic PTSD among those who were directly exposed. A wide range of variables that had been identified in previous meta-analyses was assessed. Five different categories of predictors were derived from the meta-analyses. These were: pre-trauma personal characteristics and experience, type of exposure, the individual’s emotional response at the time of the trauma, social support and subsequent long-term physical health or financial problems. While all of these categories were significant predictors, the amount of variance explained by each was modest (between 4% and 27%). Combining them improved prediction, accounting for 42% of the variance. However, this figure is still considerably less than the 63% of variance explained by psychological variables derived from Ehlers and Clark’s [18] cognitive model of PTSD. The superiority of the psychological model over general personal and trauma variables in predicting PTSD following the bombing is in line with previous research that focused on motor vehicle accidents, assaults, the Sri Lanka tsunami or emergency workers [6,17,28,29,33–35] and had considerable practical significance. Each of the psychological variables could, in principle, be modified through psychological treatment. It was therefore decided in 1999 to set up a community treatment service in Omagh that would be open to all victims of the bomb and would provide access to a cognitive therapy programme that would specifically focus on the psychological variables identified in this study. Measures of PTSD symptoms were given at each session in order to ensure that treatment response could be assessed even if patients discontinued therapy unexpectedly. A report [36] of the first 91 individuals who received treatment showed that cognitive therapy was associated with large reductions in PTSD and other symptoms, the magnitude of which was comparable to that obtained in university based randomized controlled trials. A subsequent randomized controlled trial [37] confirmed these findings with individuals with PTSD as a consequence of Omagh bomb and other conflict related traumatic events.

The findings reported here were made available to the teams planning the treatment response to the 2005 London bombings and influenced the way cognitive therapy was delivered as part of the successful screen and treatment programme [38]. Teams planning the treatment response to other bombings, including the 2011 Oslo City and the Boston Marathon bombs, may find the results similarly helpful.

A final aim of the study was to investigate the possible helpful or harmful effects of media coverage and to determine the extent to which Omagh residents felt that community cohesion had been affected by the bombing. In several studies media coverage has been found to be associated with PTSD symptom levels [11,19,20]. Interestingly in our study, many more people felt the media coverage was helpful, rather than harmful, in enabling them to come to terms with the event. This is a reassuring finding, especially as there was considerable concern at the time that the continuous flow of media reporting, which included the frequent showing on television of an amateur video recording of the immediate aftermath at the bomb scene, might have negative re-traumatizing effects [20]. However, 9% of people who were directly exposed to the bombing and associated events reported that they thought that the media coverage harmed their recovery. Future research could helpfully focus on such individuals in order to help the media identify ways in which their coverage could be modified. Like the findings on media coverage, the findings for community cohesion were also reassuring. Bombings are intended to damage communities and the importance of community and social responses to such events in conjunction with effective therapies for psychiatric problems has been reported in other studies [39,40]. One out of ten individuals who were directly exposed did report feeling less a part of the community following the bombing. However, a larger proportion (4 out of ten) reported feeling more a part of the community, with the rest reporting no change. Future studies may wish to consider how community cohesion specifically links to social support which has been identified as a factor associated with PTSD [14]. A number of factors are likely to have contributed to community cohesion in Omagh. Firstly, the age and gender profile of the victims. Many were mothers or children. People from both of the main ethno-religious groups in Northern Ireland were affected, as were visitors from the Republic of Ireland and other European countries. Also, this event was the worst in the recent history of the Northern Ireland conflict, with highest loss of life in a single incident, and unexpected as it occurred in a period of perceived peace following the signing of The Belfast Agreement. These characteristics may help explain the huge expression of empathy, solidarity and support offered from within and without the Omagh area. World leaders visited, music and sports celebrities performed but equally important, thousands of ordinary people attended vigils and memorial events affirming more positive dimensions of human behaviour after a human inflicted destructive event [39]. In addition, an active integrated network of voluntary, faith and community groups had already existed in Omagh and was highly active after the tragedy. Finally, as recommended in disaster management literature [40] after the bombing an inter-agency forum was established to discuss the impact, co-ordinate the required responses and facilitate the many support activities and memorial events. All of these factors may have helped to reduce the divisive impact of the incident on the adult population and retained a greater sense of community cohesion within the local community.

### Limitations

Our study has several limitations. As is often the case with postal surveys, the overall response rate was low. Although the response rate among individuals who were directly exposed to bombing related events was higher at around 40%, the fact that a substantial number of individuals did not reply means that the absolute rates of PTSD and other caseness that are reported here need to be interpreted with caution. While we cannot rule out the possibility that the response rate will have influenced other findings, it seems less likely that it would be an issue for our findings comparing PTSD versus other symptoms or identifying predictors of PTSD. Other limitations include the cross-sectional nature of the study and reliance on self-report measures. The timing of the study was deliberately chosen to identify individuals with chronic PTSD as this was considered the most serious problem for planning clinical services. However, this means that we were not able to identify to identify individuals with Acute Stress Disorder or to differentiate between immediate and delayed onset PTSD.

## References

[1] American Psychiatric Association (1994) Diagnostic and Statistical Manual of Mental Disorders (4th ed.) (DSM–IV).. Washington, DC: APA.

[2] NICE (2005) The management of PTSD in adults and children in primary and secondary care. Guideline26. London: National Institute for Clinical Evidence & National Collaborating Centre for Mental Health.

[3] Kessler RC, Sonnega A, Bromet E, Hughes M, Nelson CB (1995) Posttraumatic stress disorder in the National Comorbidity Survey. Arch Gen Psychiatry 52: 1048–1060. doi:10.1001/archpsyc.1995.03950240066012. PubMed: 7492257. 7492257 10.1001/archpsyc.1995.03950240066012

[4] Creamer M, Burgess P, McFarlane AC (2001) Post-traumatic stress disorder: Findings from the Australian national survey of mental health and well-being. Psychol Med, 31: 1237–1247. PubMed: 11681550. 11681550 10.1017/s0033291701004287

[5] Kessler RC, Chiu WT, Demler O, Merikangas KR, Walters EE (2005) Prevalence, severity, and comorbidity of 12-month DSM-IV disorders in the National Comorbidity Survey Replication. Arch Gen Psychiatry 62: 590–592. doi:10.1001/archpsyc.62.6.590. PubMed: 15939836. 15939839 10.1001/archpsyc.62.6.617 PMC2847357

[6] Galea A, Ahern J, Resnick H, Kilpatrick D, Bucuvalas M et al. (2002) Psychological sequelae of the September 11 terrorist attacks in New York City. N Engl J Med 346: 982-987. doi:10.1056/NEJMsa013404. PubMed: 11919308. 11919308 10.1056/NEJMsa013404

[7] Rothbaum BO, Foa EB, Riggs DS, Murdock TB, Walsh W (1992) A prospective examination of posttraumatic stress disorder in rape victims. J Trauma Stress 5: 455–475. doi:10.1002/jts.2490050309.

[8] Stein MB, Walker JR, Hazen AL, Forde DR (1997) Full and partial posttraumatic stress disorder: findings from a community survey. Am J Psychiatry 154: 1114–1119. PubMed: 9247398. 9247398 10.1176/ajp.154.8.1114

[9] Bunting BP, Ferry FR, Murphy SD, O’Neill SM, Bolton D (2013) Trauma associated with Civil Conflict and Posttraumatic Stress Disorder: Evidence from the Northern Ireland Study of Health and Stress. J Trauma Stress 26: 134-141. doi:10.1002/jts.21766. PubMed: 23417880. 23417880 10.1002/jts.21766

[10] Breslau N, Davis GC, Andreski P, Peterson E (1991) Traumatic events and posttraumatic stress disorder in an urban population of young adults. Arch Gen Psychiatry 48: 216-222. doi:10.1001/archpsyc.1991.01810270028003. PubMed: 1996917. 1996917 10.1001/archpsyc.1991.01810270028003

[11] Schlenger WE, Caddell JM, Ebert L, Jordan BK, Rourke KM et al. (2002) Psychological reactions to terrorist attacks: findings from the National Study of Americans’ Reactions to September 11. JAMA 288: 581–588. doi:10.1001/jama.288.5.581. PubMed: 12150669. 12150669 10.1001/jama.288.5.581

[12] Grieger TA, Waldrep DA, Lovasz MM, Ursano RJ (2005) Follow up of Pentagon employees two years after the terrorist attack of September 11 2001. Psychiatr Serv 56: 1374-1378. doi:10.1176/appi.ps.56.11.1374. PubMed: 16282255. 16282255 10.1176/appi.ps.56.11.1374

[13] Freh FM, Chung M, Dallos R (2013) In the shadow of terror: Posttraumatic stress and psychiatric co-morbidity following bombing in Iraq: The role of shattered world assumptions and altered self-capacities. J Psychiatr Res 47: 215-225. doi:10.1016/j.jpsychires.2012.10.008. PubMed: 23186645. 23186645 10.1016/j.jpsychires.2012.10.008

[14] Brewin CR, Andrews B, Valentine JD (2000) Meta-analysis of risk factors for post-traumatic stress disorder in trauma-exposed adults. J Consult Clin Psychol 68: 748–766. doi:10.1037/0022-006X.68.5.748. PubMed: 11068961. 11068961 10.1037//0022-006x.68.5.748

[15] Ozer EJ, Best SR, Lipsey TL, Weiss DS (2003) Predictors of post-traumatic stress disorder and symptoms in adults: A meta-analysis. Psychol Bull 129: 52–73. doi:10.1037/0033-2909.129.1.52. PubMed: 12555794. 12555794 10.1037/0033-2909.129.1.52

[16] Ehring T, Ehlers A, Glucksman E (2006) Contribution of cognitive factors to the prediction of posttraumatic stress disorder, phobia and depression after road traffic accidents. Behav Res Ther, 44: 1699-1716. doi:10.1016/j.brat.2005.11.013. PubMed: 16460669. 16460669 10.1016/j.brat.2005.11.013

[17] Ehring T, Ehlers A, Glucksman E (2008) Do cognitive models help in predicting the severity of post-traumatic stress disorder, phobia and depression after motor vehicle accidents. A prospective longitudinal study. J Consult Clin Psychol 76: 219-230. doi:10.1037/0022-006X.76.2.219. PubMed: 18377119. 18377119 10.1037/0022-006X.76.2.219 PMC2672053

[18] Ehlers A, Clark DM (2000) A cognitive model of posttraumatic stress disorder. Behav Res Ther 38: 319–345. doi:10.1016/S0005-7967(99)00123-0. PubMed: 10761279. 10761279 10.1016/s0005-7967(99)00123-0

[19] Pfefferbaum B, Seale TW, McDonald NB, Brandt EN Jr, Rainwater SM et al. (2000) Posttraumatic stress two years after the Oklahoma City bombing in youth geographically distant from the explosion. Psychiatry 63: 358–370. PubMed: 11218559. 11218559 10.1080/00332747.2000.11024929

[20] Ahern J, Galea S, Resnick H, Kilpatrick D, Bucuvalas M et al. (2002) Television images and psychological symptoms after the September 11 terrorist attacks. Psychiatry 65: 289–300. PubMed: 12530330. 12530330 10.1521/psyc.65.4.289.20240

[21] Foa EB, Cashman L, Jaycox L, Perry K (1997) The validation of a self-report measure of posttraumatic stress disorder: The posttraumatic diagnostic scale. Psychol Assess 9: 445–451. doi:10.1037/1040-3590.9.4.445.

[22] Ehring T, Kleim B, Clark DM, Foa EB, Ehlers A (2007) Screening for posttraumatic stress disorder: what combination of symptoms predicts best? J Nerv Ment Dis 195: 1004-1012. doi:10.1097/NMD.0b013e31815c1999. PubMed: 18091194. 18091194 10.1097/NMD.0b013e31815c1999

[23] Ehlers A, Grey N, Wild J, Stott R, Linnes S, Deale A, Handley R, Albert I, Cullen D, Hackmann A, Manley J, McManus F, Brady F, Salkovskis P, Clark DM (in press) Implementation of cognitive therapy for PTSD in routine clinical care: effectiveness and moderators of outcome in a consecutive sample. Behav Res Ther. 10.1016/j.brat.2013.08.006 PMC3897916 24076408

[24] Goldberg D, Williams PA (1988) User’s guide to the general health questionnaire. Windsor: NFER.

[25] Foa EB, Ehlers A, Clark DM, Tolin DM, Orsillo SM (1999) The Posttraumatic Cognitions Inventory (PTCI): Development and validation. Psychol Assess, 11: 303–314. doi:10.1037/1040-3590.11.3.303.

[26] Halligan SL, Michael T, Clark DM, Ehlers A (2003) Posttraumatic stress disorder following assault: the role of cognitive processing, trauma memory, and appraisals. J Consult Clin Psychol 71: 419-431. doi:10.1037/0022-006X.71.3.419. PubMed: 12795567. 12795567 10.1037/0022-006x.71.3.419

[27] Michael T, Ehlers A, Halligan SL, Clark DM (2005) Unwanted memories of assault: What intrusion characteristics predict PTSD? Behav Res Ther, 43: 613-628. doi:10.1016/j.brat.2004.04.006. PubMed: 15865916. 15865916 10.1016/j.brat.2004.04.006

[28] Clohessy S, Ehlers A (1999) PTSD symptoms, response to intrusive memories, and coping in ambulance service workers. Br J Clin Psychol 38: 251-265. doi:10.1348/014466599162836. PubMed: 10532147. 10532147 10.1348/014466599162836

[29] Murray J, Ehlers A, Mayou RA (2002) Dissociation and posttraumatic stress disorder: Two prospective studies of road traffic accident survivors. Br J Psychiatry 180: 363–368. doi:10.1192/bjp.180.4.363. PubMed: 11925361. 11925361 10.1192/bjp.180.4.363

[30] Galea S, Vlahov D, Resnick H, Ahern J, Susser E et al. (2003) Trends of Probable Post-Traumatic Stress Disorder in New York City after the September 11 Terrorist Attacks. Am J Epidemiol 158: 514-524. doi:10.1093/aje/kwg187. PubMed: 12965877. 12965877 10.1093/aje/kwg187

[31] North CS, Nixon SJ, Shariat S, Mallonee S, McMillen JC et al. (1999) Psychiatric disorders among survivors of the Oklahoma City bombing. JAMA 282: 755-762. doi:10.1001/jama.282.8.755. PubMed: 10463711. 10463711 10.1001/jama.282.8.755

[32] Green B, Grace M, Lindy J, Gleser GC, Leonard AC, Kramer TL (1990) Buffalo Creek survivors in the second decade: comparison with unexposed and nonlitigant groups. J Appl Soc Psychol 20: 1033-1050. doi:10.1111/j.1559-1816.1990.tb00388.x.

[33] Kleim B, Ehlers A, Glucksman E (2007) Prediction of chronic posttraumatic stress disorder after assault. Psychol Med 37: 1457-1468. PubMed: 17588274. 17588274 10.1017/S0033291707001006 PMC2829994

[34] Kleim B, Ehlers A, Glucksman E (2012) Investigating cognitive pathways to psychopathology: Predicting depression and posttraumatic stress disorder from early responses after assault. Psychological Trauma Theory Res Practice And Policy 4: 527-537. doi:10.1037/a0027006. PubMed: 23002418. 10.1037/a0027006 PMC3444173 23002418

[35] Lommen MJJ, Sanders AJML, Buck N, Arntz A (2009) Psychosocial predictors of chronic post-traumatic stress disorder in Sri Lankan tsunami survivors. Behav Res Ther 47: 60-65. doi:10.1016/j.brat.2008.10.009. PubMed: 19013551. 19013551 10.1016/j.brat.2008.10.009

[36] Gillespie K, Duffy M, Hackmann A, Clark DM (2002) Community based cognitive therapy in the treatment of post-traumatic stress disorder following the Omagh bomb. Behav Res Ther 40: 345-357. doi:10.1016/S0005-7967(02)00004-9. PubMed: 12002894. 12002894 10.1016/s0005-7967(02)00004-9

[37] Duffy M, Gillespie K, Clark DM (2007) Posttraumatic stress disorder in the context of terrorism and other civil conflict in Northern Ireland: randomized controlled trial. BMJ 334: 147-150. doi:10.1136/bmj.39043.685775.F7. 10.1136/bmj.39021.846852.BE PMC1885307 17495988

[38] Brewin CR, Scragg P, Robertson M, Thompson M, Ardenne P, Ehlers A (2008) Promoting mental health following the London bombings: a screen and treat approach. J Trauma Stress 21: 3-8. doi:10.1002/jts.20310. PubMed: 18302178. 18302178 10.1002/jts.20310 PMC2958457

[39] Norwood AE, Ursano RJ, Fullerton CS (2000) Disaster psychiatry: Principles and practice. Psychiatr Q 71: 207-226. doi:10.1023/A:1004678010161. PubMed: 10934746. 10934746 10.1023/a:1004678010161

[40] Ursano RJ, McCaughey BC, Fullerton CS (1994) Individual and community responses to trauma and disaster: The structure of human chaos. Cambridge, England: Cambridge University Press.

